# Supplementary material for: Genome-to-genome analysis reveals associations between human and mycobacterial genetic variation in tuberculosis patients from Tanzania
Source: BMC Med Genomics. 2025 Jun 2;18:99. doi: 10.1186/s12920-025-02164-x (PMC12131348; doi:10.1186/s12920-025-02164-x)

Supplementary Tables and Figures

Supplementary Table 1

Comparison of human tribes and M. tuberculosis lineages between patients with paired human and bacterial genomic data against those without (Excluded). P-values based on Chi-squared test.

	Excluded (N=2023)	Human-M.tb Pairs (N=1000)	P-value
**Patient Tribe**			
Chaga	71 (4 %)	48 (5 %)	0.0858
Makonde	147 (7 %)	77 (8 %)	
Matumbi	74 (4 %)	40 (4 %)	
Mwera	107 (5 %)	56 (6 %)	
Ndengereko	343 (17 %)	138 (14 %)	
Ngindo	77 (4 %)	27 (3 %)	
Ngoni	62 (3 %)	39 (4 %)	
Zaramo	264 (13 %)	114 (11 %)	
Other	878 (43 %)	461 (46 %)	
***M.tb* Lineage**			
L1	65 (15 %)	144 (15 %)	0.0926
L2	25 (6 %)	78 (8 %)	
L3	220 (50 %)	412 (43 %)	
L4	133 (30 %)	317 (33 %)	
Missing	1580	49	

Supplementary Table 2

Filters recommended by Illumina to exclude poor quality probes. Minor allele frequency (MAF) based filters were not applied to X-Chr probes.
Adapted from:
https://www.illumina.com/Documents/products/technotes/technote_infinium_genotyping_data_analysis.pdf

Metric(s)	Threshold for exclusion
Rep Errors	> 2
PPC Errors	> 2
Cluster Sep	≤ 0.3
AA R Mean	≤ 0.2
AB R Mean	≤ 0.2
BB R Mean	≤ 0.2
10%_GC_Score	≤ 0.3
Hex Excess	> 0.2
A/B_Freq	≥ 0.4
AB T Mean	(< 0.2) or (> 0.8)
A/A_Freq = 1	
AA T Mean	> 0.3
AA T Dev	> 0.06
B/B_Freq = 1	
BB T Mean	< 0.7
BB T Dev	> 0.06
A/A_Freq or B/B_Freq = 0	
AB T Dev	> 0.05
A/B_Freq = 0	
MAF	> 0
0.998 > Call_Freq > 0.990	
MAF	< 0.05

Supplementary Table 3

Prevalence (allele frequencies) of associated human and *M.tb* variants within self-reported tribes, grouped based on their geographic origin within Tanzania.

**Tribe Region**	**N**	**rs12151990**	**Rv2348 I101M**	**rs75769176**	**fixA T67M**	**DRB1 H96E**	**EsxB E68K**
Dar es Salaam and Pwani	110	0.088	0	0.044	0.018	0.066	0.031
Kilimanjaro	73	0.11	0.014	0.082	0.041	0.074	0.015
Kilwa District	56	0.12	0.036	0.054	0.018	0.056	0.037
Pwani	140	0.079	0.021	0.059	0.034	0.043	0.0072
Tanga	56	0.14	0.036	0.027	0.018	0.039	0
Multiple	120	0.074	0.025	0.05	0.025	0.048	0.018
Other	440	0.087	0.016	0.069	0.018	0.054	0.015

Supplementary Table 4

Association between Rv2348c I101M and host variant (rs12151990), covariates, and patient characteristics based on logistic regression. The minor allele of rs12151990 (T) was encoded as the effect allele.

	log(OR)	SD	P
rs12151990	1.73	0.26	**4.67e-11**
**Covariates**			
Sex	-7.7e-3	9.2e-3	0.40
Human PC1	-0.03	0.16	0.86
Human PC2	0.14	0.16	0.40
Human PC3	-0.02	0.16	0.92
**Patient Characteristics**			
Age	-3.8e-4	4.2e-3	0.93
BMI	2.3e-3	4.2e-3	0.59
Smoker	3.6e-3	9.9e-3	0.72
HIV Positive	6.9e-3	0.01	0.55

Supplementary Table 5

Association between FixA T67M and host variant (rs75769176), covariates, or patient characteristics based on logistic regression. The minor allele of rs75769176 (G) was encoded as the effect allele.

	log(OR)	SD	p
rs75769176	1.9	0.29	**6.28e-11**
**Covariates**			
Sex	5.8e-4	0.01	0.95
Human PC1	0.27	0.18	0.14
Human PC2	-0.56	0.18	**2.3e-3**
Human PC3	-0.25	0.18	0.165
**Patient Characteristics**			
Age	-2.8e-3	4.7e-3	0.55
BMI	8.1e-3	4.7e-3	0.09
Smoker	-3.1e-3	0.01	0.78
HIV Positive	-0.01	0.01	0.41

Supplementary Table 6

Association between clinical measures and genome-to-genome (G2G) associated variants. Ordinal logistic regression was used for Bandim TB Score and linear regression was used for X-ray score and GeneXpert Ct-value. Estimates correspond to beta for linear regression and log odds for ordinal regression. Covariates were selected using bidirectional stepwise selection.

Dependent Variable	Covariates	Independent Variable	p	Estimate (SE)
Bandim TB score	Cough_Duration + Sex + Smoking + HIV Status	rs12151990	0.76	0.04 (0.13)
		Rv2348c I101M	0.78	0.12 (0.41)
		**rs75769176**	**0.04**	**-0.34 (0.17)**
		FixA T67M	0.13	-0.58 (0.38)
X-ray score	Age + Sex + HIV Status	rs12151990	0.22	0.13 (0.10)
		Rv2348c I101M	0.21	0.39 (0.31)
		rs75769176	0.87	-0.02 (0.12)
		FixA T67M	0.76	0.09 (0.30)
Ct value	HIV Status + Cough Duration + Smoking	rs12151990	0.86	0.02 (0.10)
		Rv2348c I101M	0.64	-0.19 (0.40)
		rs75769176	0.38	0.12 (0.13)
		FixA T67M	0.09	0.48 (0.29)

Supplementary Table 7

Human variants previously identified to be associated with susceptibility for developing active TB. The top G2G association for each human variant is displayed. If the query variant was not common within our cohort, proxy variant indicates a human variant within ±5kb that displays the strongest association with a *M.tb* variant.

Study	Human Variant (Gene)	Proxy Variant	*M.tb* Variant	P	OR
**Associations with TB susceptibility**					
Thye et al, 2012(13) Chimusa et al, 2014(14)	rs2057178 (*WT1*)		Rv0178:p.Asp150Glu	1.8e-2	0.6
Thye et al, 2010(15)	rs4331426		hsdM:p.Lys483Glu	5.5e-2	1.7
Curtis et al, 2015(16)	rs10956514 (*ASAP1*)		eccE1:p.Phe113fs	4.4e-2	1.4
	rs4733781 (ASAP1)		pyrH:p.Ala247Val	3.3e-3	2.4
Zheng et al, 2018(17)	rs12437118 (*ESRRB*)		Rv2078:p.Gly74Asp	1.8e-3	0.4
	rs6114027 (*TGM6*)		fixA:p.Thr67Met	4.3e-4	0.3
Luo et al, 2019(18)	rs73226617 (*ATP1B3*)	rs4683619	fixA:p.Thr67Met	1.8e-3	0.4
Kerner et al, 2019(19)	rs34536443 (*TYK2*)	rs203999	PE35:p.Met4fs	1.7e-3	0.4
Quistrebert et al, 2021(20)	rs17155120 (C10orf90/ADAM21)		Rv2425c:p.Met289Ile	7.6e-3	1.6
Qi et al, 2017(21)	rs4240897 (*MFN2*)		Rv2963:p.Gly165fs	9.5e-5	1.4
Sobota et al, 2016(22)	rs4921437 (*IL12B*)		ltp1:p.Ser6fs	8.2e-4	2.4

Supplementary Table 8

Human variants previously identified to be associated with lineages or clades of *M.tb*. The top G2G association for each human variant is displayed. If the query variant was not common within our cohort, proxy variant indicates a human variant within ±5kb that displays the strongest association with a *M.tb* variant.

Study	Human Variant (Gene)	Proxy Variant	*M.tb* Variant	P	OR
**Associations with *M.tb* Lineage / Clade**					
McHenry et al, 2020(36)	rs17235409 (*SLC11A1*)	rs146731249	hsdM:p.Lys483Glu	5.0e-6	4.8
McHenry et al, 2021(35)	rs114945555 (*PPIAP22*)		Rv3916c:p.Leu221Val	6.6e-2	2.5
Luo et al, 2022(33)	rs3130660 (*FLOT1*)	rs113903830	Rv2179c:p.Glu30Lys	6.1e-3	1.7
Müller et al, 2021(37)	rs529920		Rv0574c:p.Tyr45His	1.3e-2	2.0
	rs41472447 (*PDZRN4*)	rs36070978	Rv0104:p.Ile56Ser	3.7e-3	3.4
Phelan et al, 2023(34)	rs267951 (*DAP*)		helZ:p.Ile727Leu	1.4e-2	0.4
	rs74875032	rs4900336	Rv2949c:p.Trp189Leu	2.2e-4	2.4
	rs60284130 (*MFAP2*)	rs56265117	Rv0435c:p.Ala61Thr	1.1e-3	0.4
	rs142600697 (*FSTL5*)	rs61381755	Rv3847:p.Ile170Thr	1.1e-5	3.9
	rs1118438		Rv0377:p.Arg302Pro	2.9e-2	0.8
	rs558237 (*RIMS3*)		Rv2134c:p.Asp192Ala	3.0e-2	0.6
	rs59441182		Rv2026c:p.Pro102Thr	2.1e-2	0.5
	rs4563899 (*CSGALNACT*)		Rv0839:p.Ala205Thr	2.4e-3	0.4

Supplementary Table 9

Experimentally validated epitopes (according to IEDB) that each *M.tb* variant maps to (with location of variant indicated in square brackets within epitope sequence). If an epitope has been experimentally validated to be restricted by an HLA-DRB1 allele, the respective alleles are indicated.

***M.tb* protein**	***M.tb* variant**	***M.tb* T cell epitope**	**IEDB experiment - human HLA-DRB1 restriction**
EspK	L39W	DTFYDRAQEYSQV[L>W]Q	N/A
EsxB	E68K	AAGTAAQAAVVRFQEAANKQKQ[E>K]LD,<br>AANKQKQ[E>K]LDEISTN,<br>AANKQKQ[E>K]LDEISTNIRQAG,<br>AANKQKQ[E>K]LDEISTNIRQAGVQYSR,<br>AAVVRFQEAANKQKQ[E>K]L,<br>AQAAVVRFQEAANKQKQ[E>K]LD,<br>[E>K]LDEISTNIRQAGVQYSR,<br>KQ[E>K]LDEISTNIRQAG,<br>RFQEAANKQKQ[E>K]LDE,<br>VRFQEAANKQKQ[E>K]LD,<br>VVRFQEAANKQKQ[E>K]L,<br>AVVRFQEAANKQKQ[E>K],<br>[E>K]LDEISTNIRQAGVQ,<br>FQEAANKQKQ[E>K]LDEI,<br>Q[E>K]LDEISTNIRQAGV	N/A<br>HLA-DRB1*04:05<br>N/A<br>N/A<br>N/A<br>N/A<br>N/A<br>HLA-DRB1*03:01,04:01,08:02,11:01,13:02<br>N/A<br>HLA-DRB1*04:01,04:04,04:05,09:01,11:01<br>N/A<br>N/A<br>N/A<br>N/A<br>N/A
Mpt70	A21T	GLAAL[A>T]VAVSPPAAAGDLVGPGCAE,<br>MKVKNTIAATSFAAAGLAAL[A>T]VAVS,<br>SFAAAGLAAL[A>T]VAVSPPAA	N/A<br>N/A<br>N/A
RimJ	R72L	G[R>L]MLPYVIEL	N/A

Supplementary Figure 1

Diagram illustrating the sample inclusion criteria for analysis based on data availability

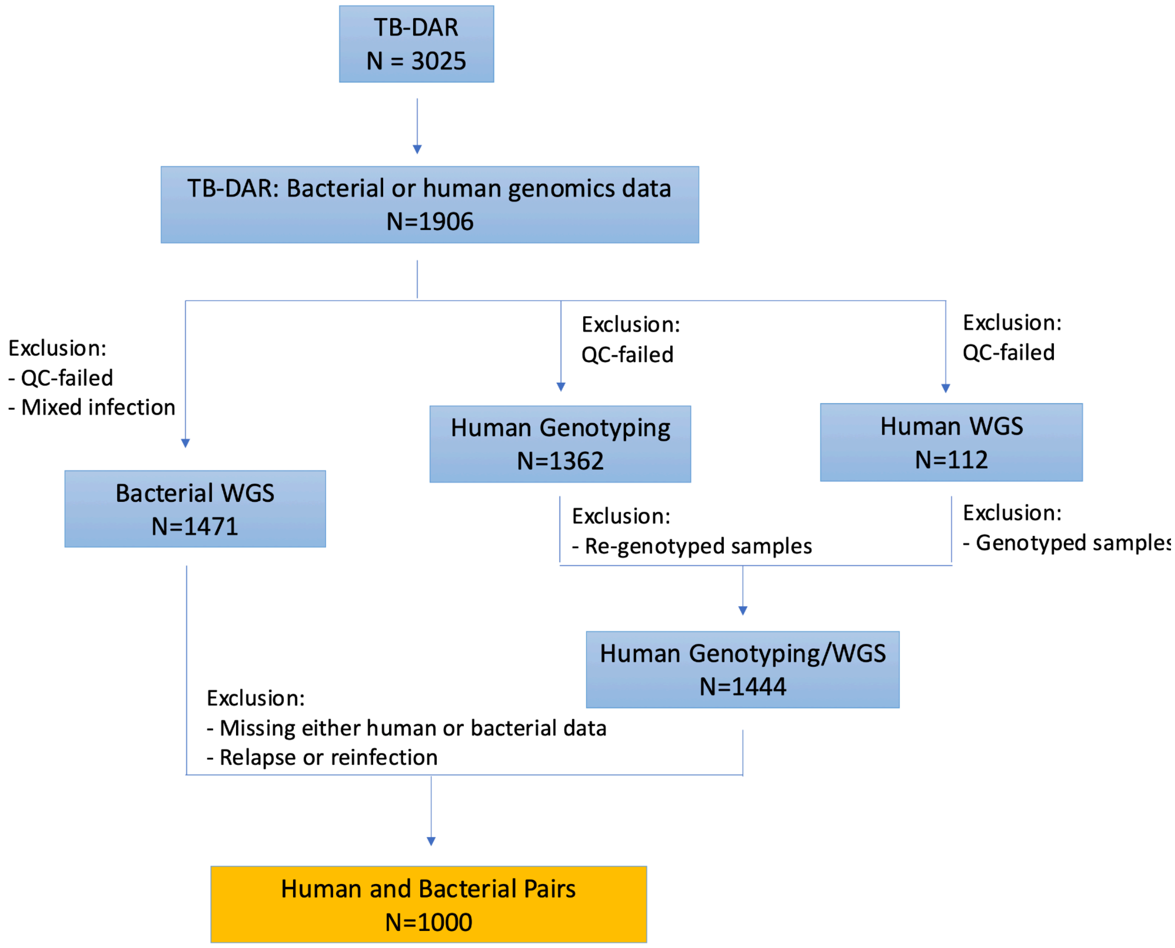

Supplementary Figure 2

A)
Top two human genetic principal components (PCs) based on a merged dataset of our cohort (TBDAR) and 1000 Genome superpopulations. A single outlier within our cohort that was not assigned to the African population is labeled. 1000 Genomes Super Populations: AFR - African, EAS - East Asian, EUR - European, SAS - South Asian.

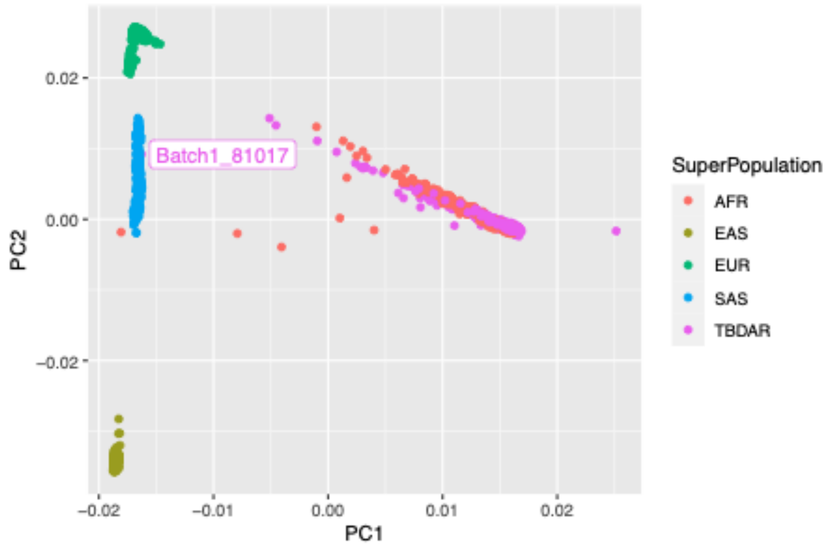

B)
Top two human genetic principal components (PCs) of our cohort. Variance explained is indicated in brackets. Colors indicate whether the sample was genotyped and imputed or whole-genome sequenced.

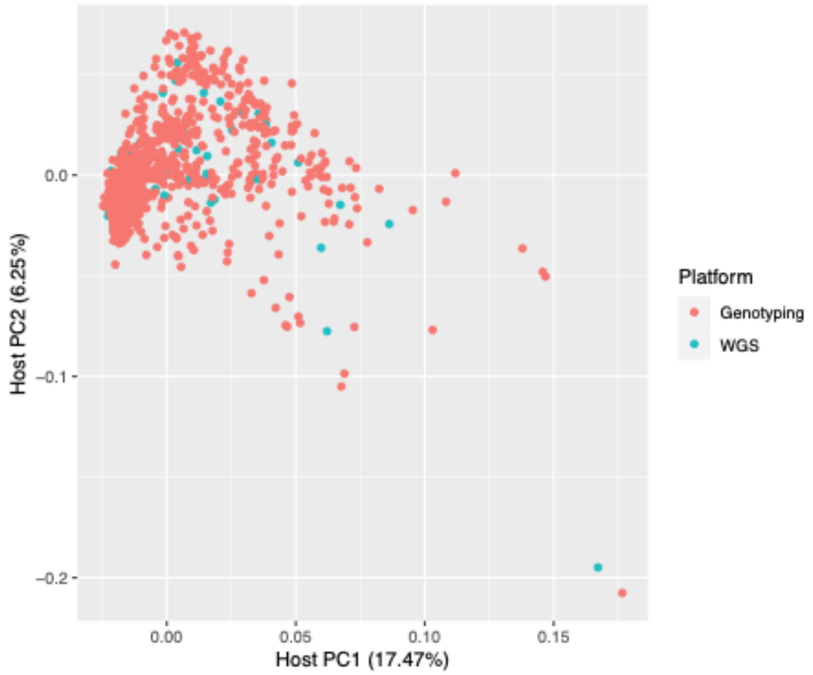

Supplementary Figure 3

Variance explained by each human genetic principal component (PC).

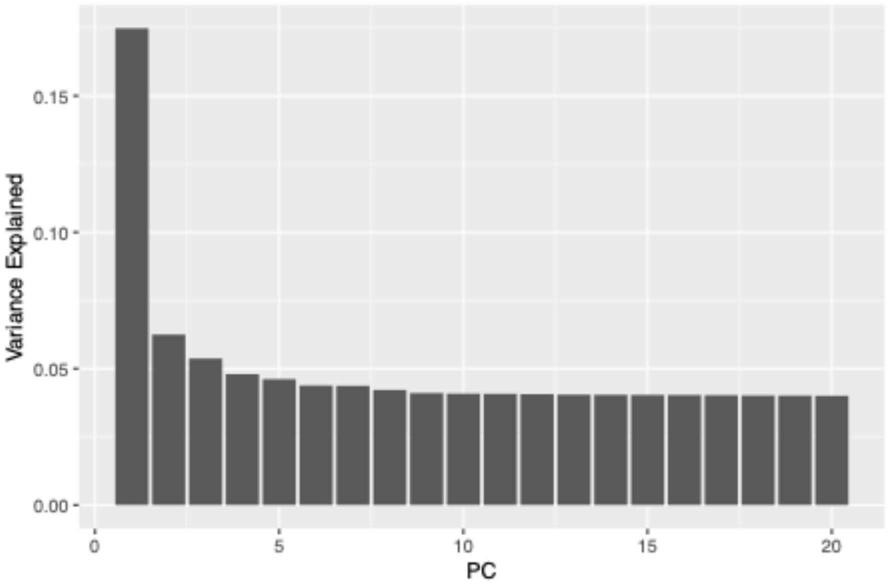

Supplementary Figure 4

Statistical power calculation assuming an alpha level that accounts for multiple testing (5e-8 / Number of independent *M.tb* variants at specific AF threshold). An additive test and true model was assumed. Statistical power is shown for each variant effect size measured by odds ratio (OR), human variant minor-allele frequency (MAF), and *M.tb* minor-allele frequency (*M.tb* AF). A *M.tb* AF threshold of 0.015 was chosen, given that for *M.tb* variants with lower frequency there is very low statistical power to detect associations with common host variants (MAF > 0.05) even under the assumption of a very high effect size (OR = 25).

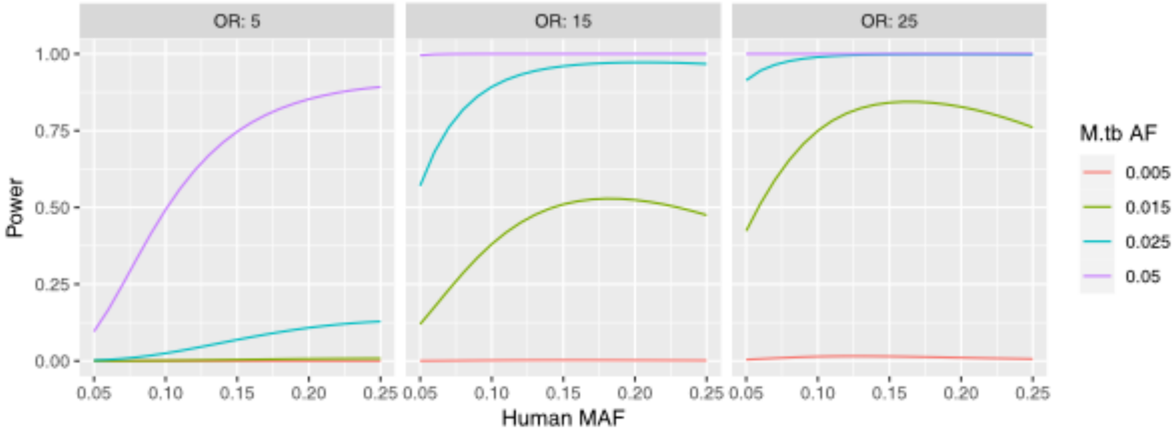

Supplementary Figure 5

Lack of association between human and *M.tb* population structure. **A)** Human PC1 - PC4, colored according to bacterial lineage **B)** Pearson correlation coefficients (r) between top 10 human PCs and *M.tb* phylogenetic principal components (pPCs).

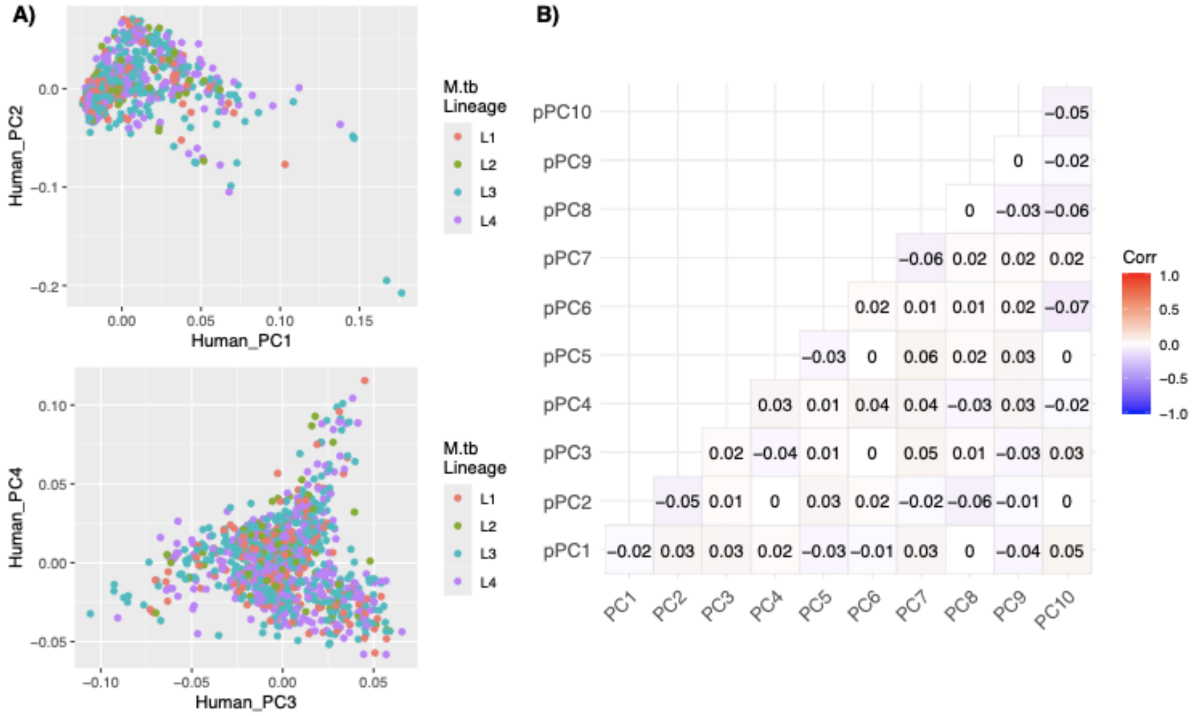

Supplementary Figure 6

Genomic inflation factor (λ) for all GWAS conducted as part of the genome-to-genome (G2G) study.

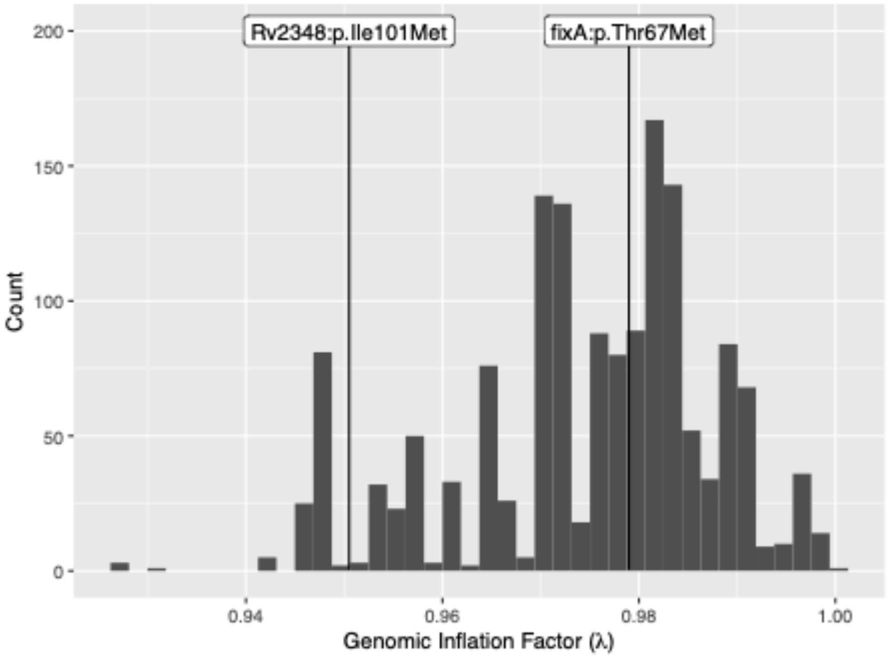

Supplementary Figure 7

M.tb variants that are in perfect correlation were grouped into variant-sets. Variant-sets of size 1 indicate independent *M.tb* variants (N = 436). Variant-sets of size greater than one (N=52) indicate groups of perfectly correlated variants. 1102 variants belong to such variant-sets.

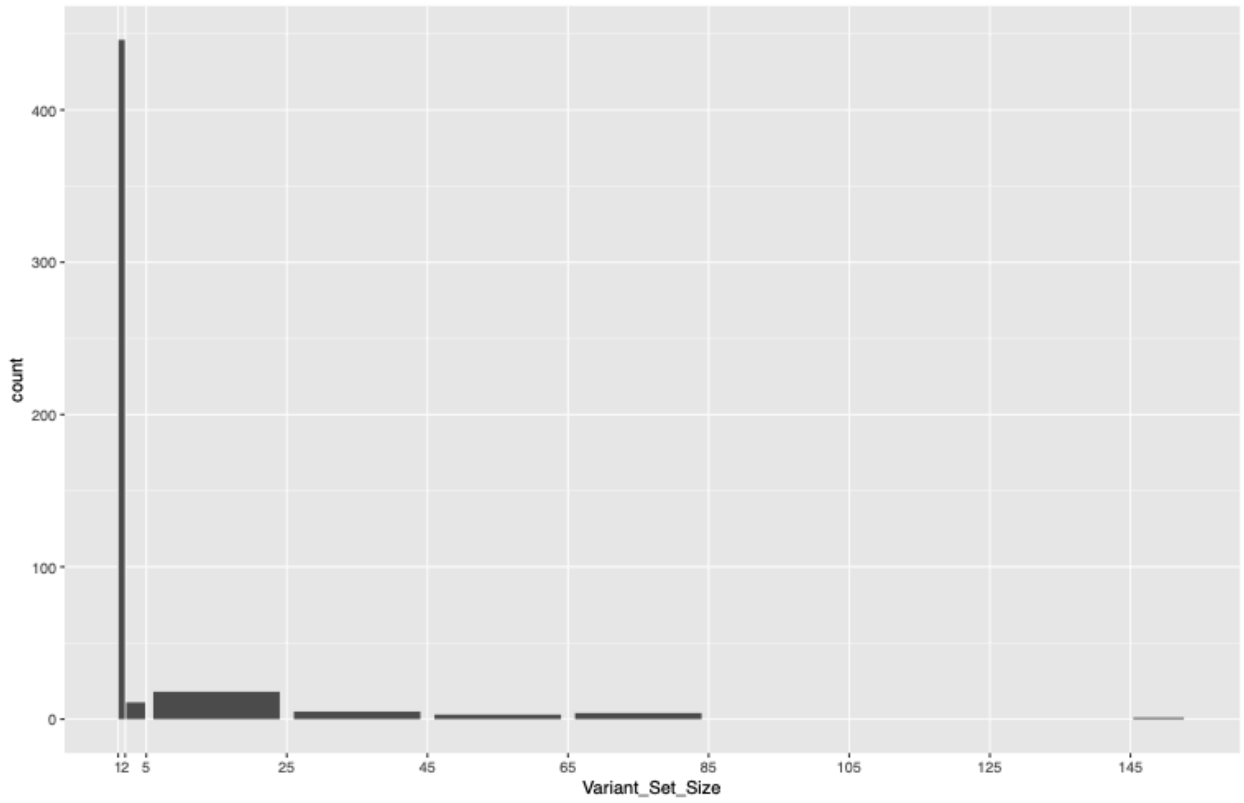

Supplementary Figure 8

Each dot represents a genome-to-genome (G2G) association between a human and *M.tb* variant, with colors indicating p-value. The two significant associations are circled.

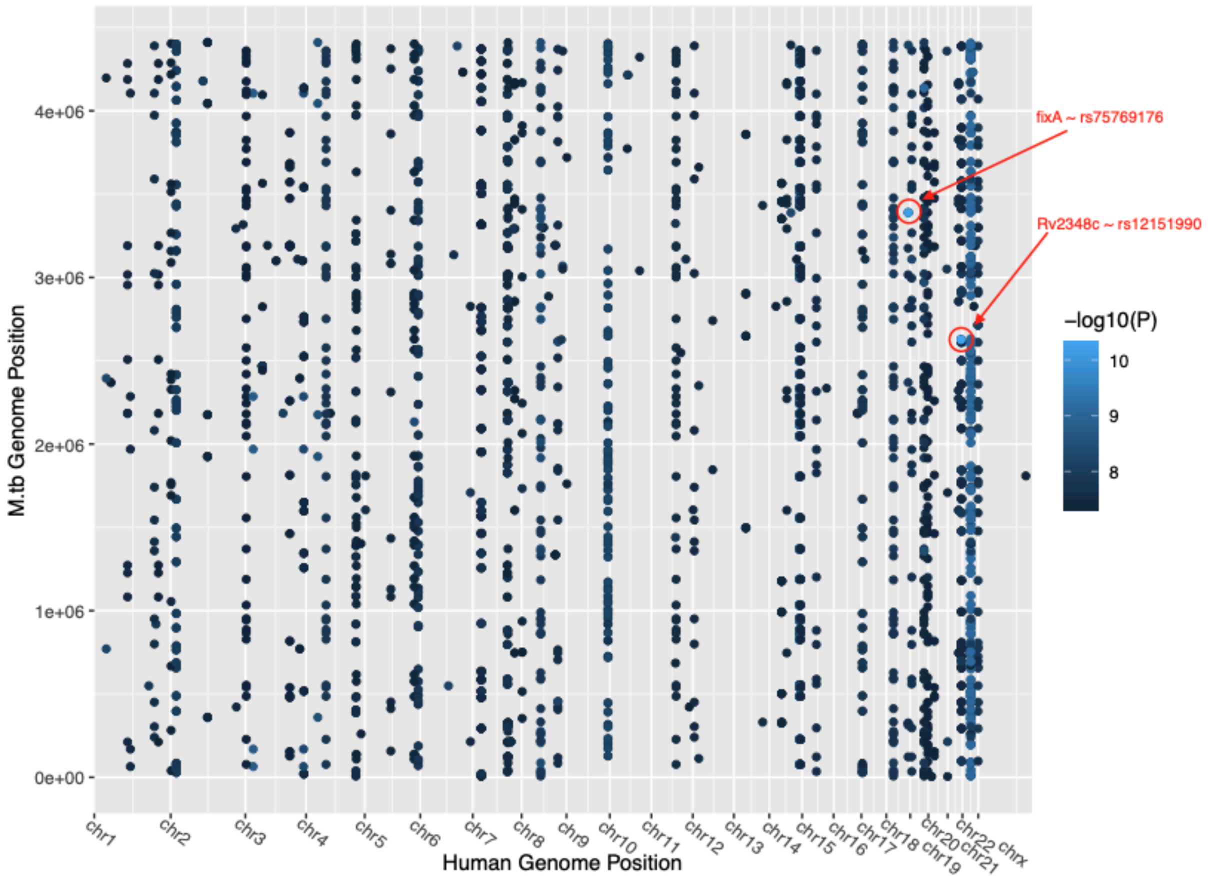

Supplementary Figure 9

Locus zoom plot of **A)** human genetic loci of association between Rv2348c I101M and rs12151990 **B)** human genetic loci of association between FixA T67M and rs75769176

A)

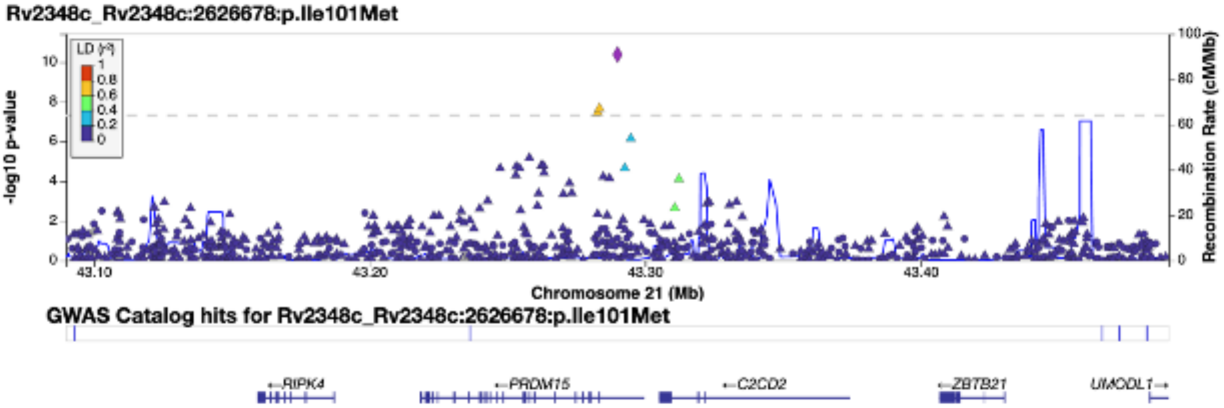

B)

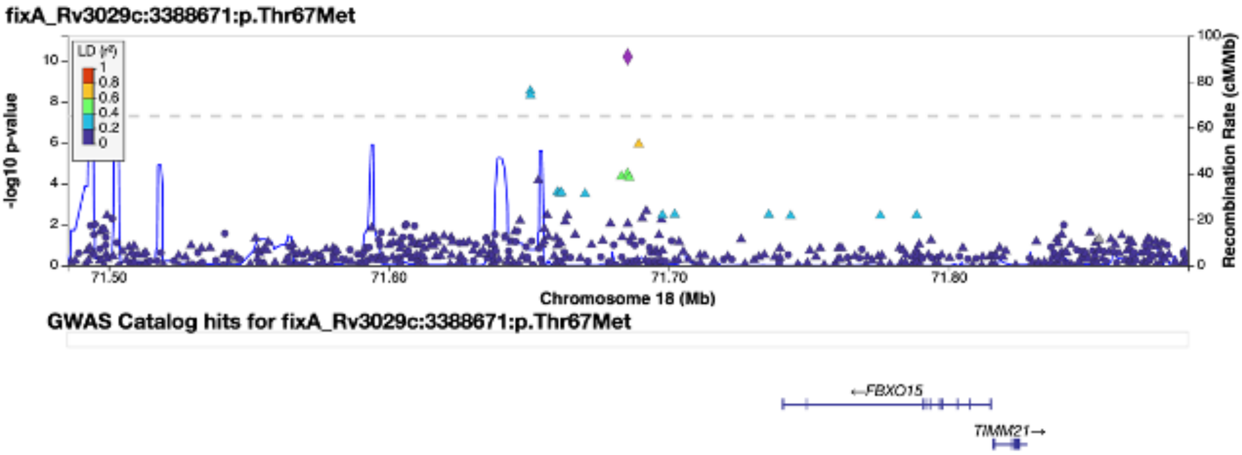

Supplementary Figure 10

Posterior probability of shared causal variant based colocalization of GWAS and eQTL signals across different tissues. Studies that include individuals with African ancestry are highlighted in green. **A)** between rs12151990 and eQTLs of *PRDM15* **B)** between rs75769176 and eQTLs of *FBXO15* **C)** between rs75769176 and eQTLs of *TIMM21*

A)

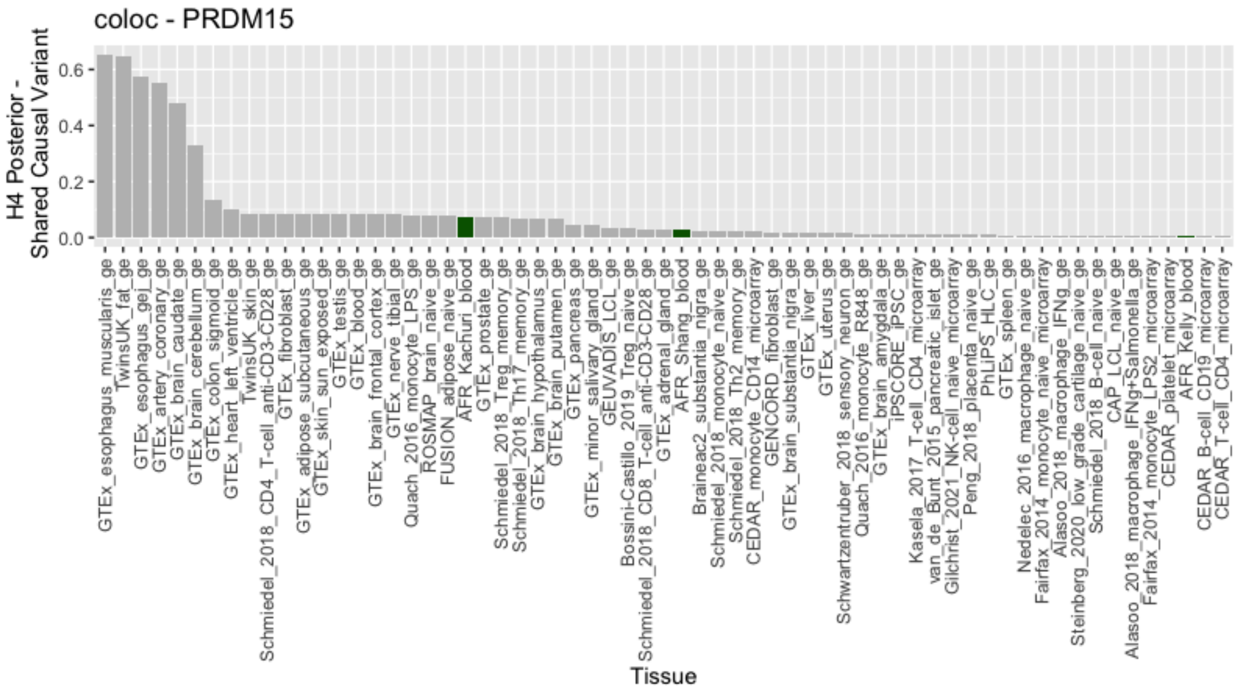

B)

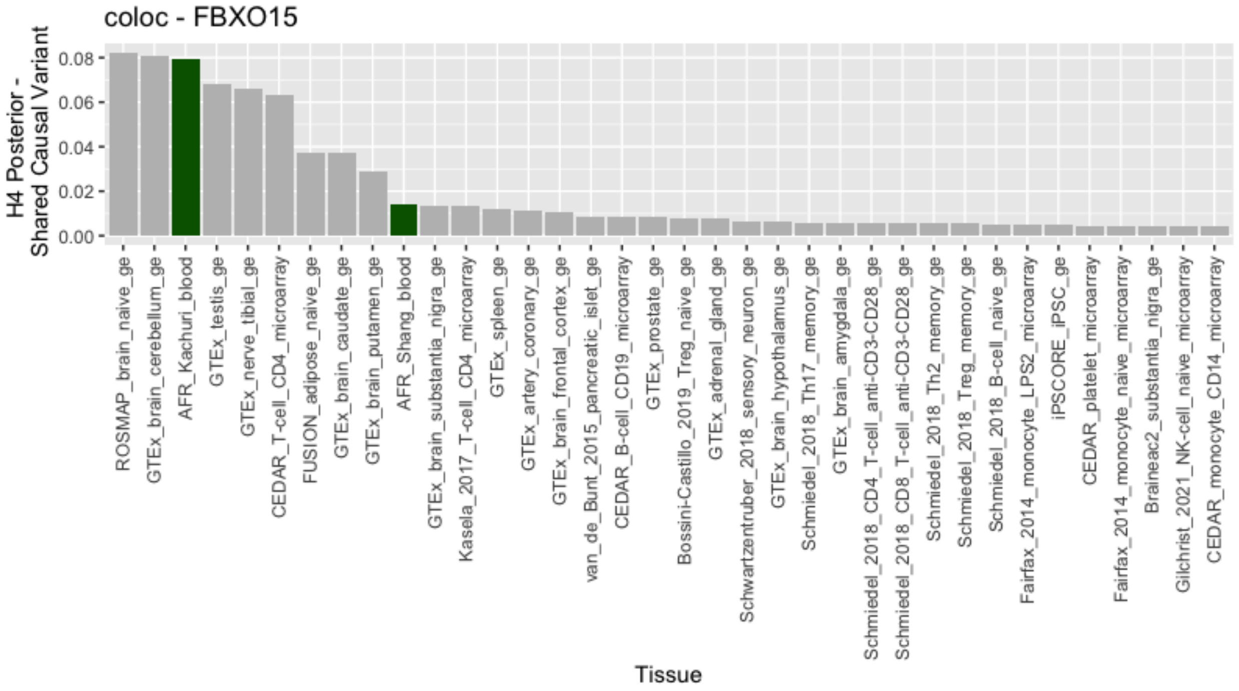

coloc - FBXO15
H4 Posterior - Shared Causal Variant
0.08
0.06
0.04
0.02
0.00
ROSMAP_brain_naive_ge
GTEx_brain_cerebellum_ge
AFR_Kachuri_blood
GTEx_testis_ge
GTEx_nerve_tibial_ge
CEDAR_T-cell_CD4_microarray
FUSION_adipose_naive_ge
GTEx_brain_caudate_ge
GTEx_brain_putamen_ge
AFR_Shang_blood
GTEx_brain_substantia_nigra_ge
Kasela_2017_T-cell_CD4_microarray
GTEx_spleen_ge
GTEx_artery_coronary_ge
GTEx_brain_frontal_cortex_ge
van_de_Bunt_2015_pancreatic_islet_ge
CEDAR_B-cell_CD19_microarray
GTEx_prostate_ge
Bossini-Castillo_2019_Treg_naive_ge
GTEx_adrenal_gland_ge
Schwartzentruber_2018_sensory_neuron_ge
GTEx_brain_hypothalamus_ge
Schmiedel_2018_Th17_memory_ge
GTEx_brain_amygdala_ge
Schmiedel_2018_CD4_T-cell_anti-CD3-CD28_ge
Schmiedel_2018_CD8_T-cell_anti-CD3-CD28_ge
Schmiedel_2018_Th2_memory_ge
Schmiedel_2018_Treg_memory_ge
Schmiedel_2018_B-cell_naive_ge
Fairfax_2014_monocyte_LPS2_microarray
iPSCORE_iPSC_ge
CEDAR_platelet_microarray
Fairfax_2014_monocyte_naive_microarray
Braineac2_substantia_nigra_ge
Gilchrist_2021_NK-cell_naive_microarray
CEDAR_monocyte_CD14_microarray
Tissue

C)

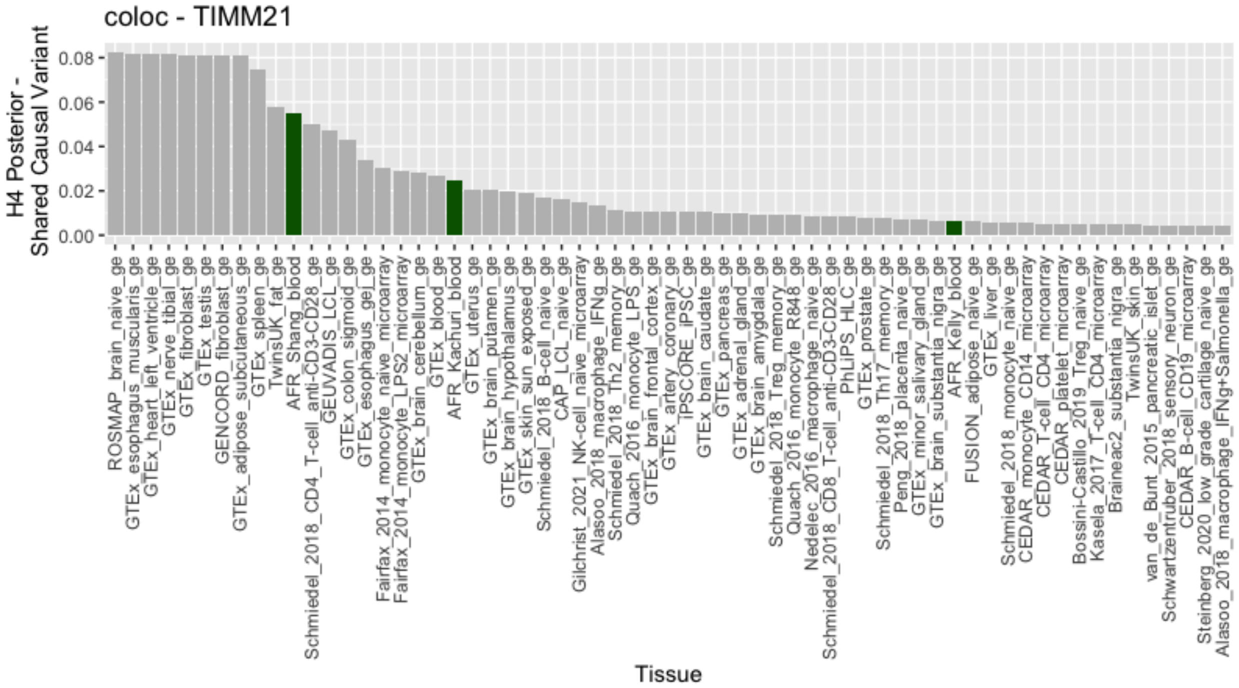

coloc - TIMM21
H4 Posterior - Shared Causal Variant
0.08
0.06
0.04
0.02
0.00
ROSMAP_brain_naive_ge
GTEx_esophagus_muscularis_ge
GTEx_heart_left_ventricle_ge
GTEx_nerve_tibial_ge
GTEx_fibroblast_ge
GTEx_testis_ge
GENCORD_fibroblast_ge
GTEx_adipose_subcutaneous_ge
GTEx_spleen_ge
TwinsUK_fat_ge
AFR_Shang_blood
Schmiedel_2018_CD4_T-cell_anti-CD3-CD28_ge
GEUVADIS_LCL_ge
GTEx_colon_sigmoid_ge
GTEx_esophagus_gej_ge
Fairfax_2014_monocyte_naive_microarray
Fairfax_2014_monocyte_LPS2_microarray
GTEx_brain_cerebellum_ge
GTEx_blood_ge
AFR_Kachuri_blood
GTEx_uterus_ge
GTEx_brain_putamen_ge
GTEx_brain_hypothalamus_ge
GTEx_skin_sun_exposed_ge
Schmiedel_2018_B-cell_naive_ge
CAP_LCL_naive_ge
Gilchrist_2021_NK-cell_naive_microarray
Alasoo_2018_macrophage_IFNg_ge
Schmiedel_2018_Th2_memory_ge
Quach_2016_monocyte_LPS_ge
GTEx_brain_frontal_cortex_ge
GTEx_artery_coronary_ge
iPSCORE_iPSC_ge
GTEx_brain_caudate_ge
GTEx_pancreas_ge
GTEx_adrenal_gland_ge
GTEx_brain_amygdala_ge
Schmiedel_2018_Treg_memory_ge
Quach_2016_monocyte_R848_ge
Nedelec_2016_macrophage_naive_ge
Schmiedel_2018_CD8_T-cell_anti-CD3-CD28_ge
PhLiPS_HLC_ge
GTEx_prostate_ge
Schmiedel_2018_Th17_memory_ge
Peng_2018_placenta_naive_ge
GTEx_minor_salivary_gland_ge
GTEx_brain_substantia_nigra_ge
AFR_Kelly_blood
FUSION_adipose_naive_ge
GTEx_liver_ge
Schmiedel_2018_monocyte_naive_ge
CEDAR_monocyte_CD14_microarray
CEDAR_T-cell_CD4_microarray
CEDAR_platelet_microarray
Bossini-Castillo_2019_Treg_naive_ge
Kasela_2017_T-cell_CD4_microarray
Braineac2_substantia_nigra_ge
TwinsUK_skin_ge
van_de_Bunt_2015_pancreatic_islet_ge
Schwartzentruber_2018_sensory_neuron_ge
CEDAR_B-cell_CD19_microarray
Steinberg_2020_low_grade_cartilage_naive_ge
Alasoo_2018_macrophage_IFNg+Salmonella_ge
Tissue

Supplementary Figure 11

Prevalence of identified *M.tb* variants in global reference set *M.tb* genomes **A)** FixA T67M in L3 **B)** Rv2348c I101M in L4

A)

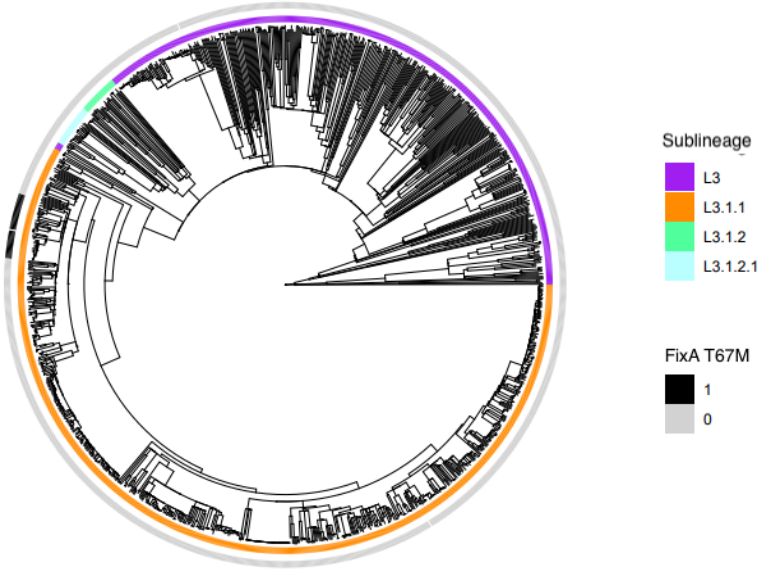

B)

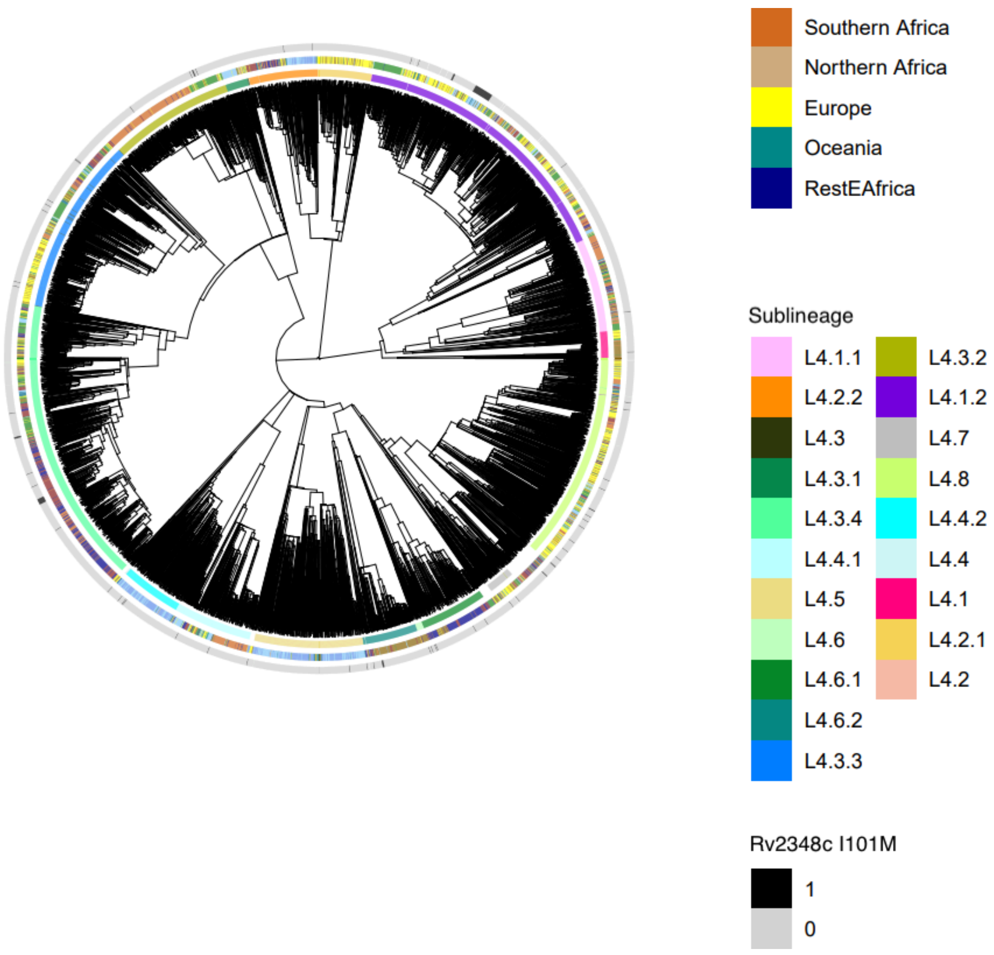

Supplementary Figure 12

Pearson correlation coefficient (r) between *M.tb* variants and human principal components (PCs)

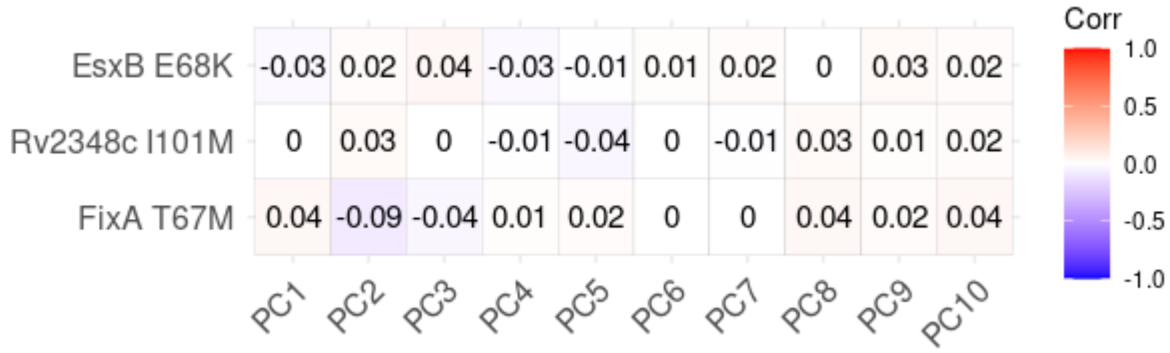

Supplementary Figure 13

Human genetic distances between patients infected with *M.tb* strains carrying the same variant (FixA T67M, Rv2348c I101M, or EsxB E68K) compared against patients from the same self-reported tribe.

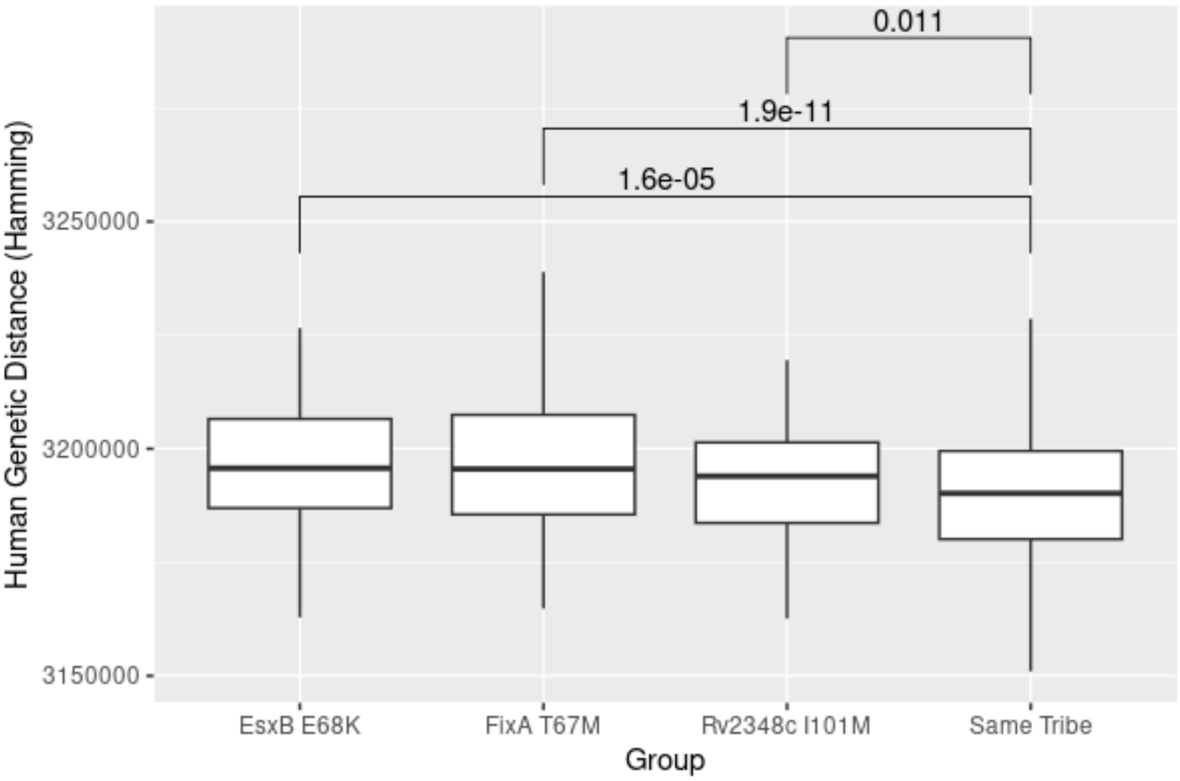

Supplementary Figure 14

Distribution of module-module density values based on permuted dataset. P-value indicates significance of a density of 0.11, corresponding to the pair of human and *M.tb* modules with the highest density.

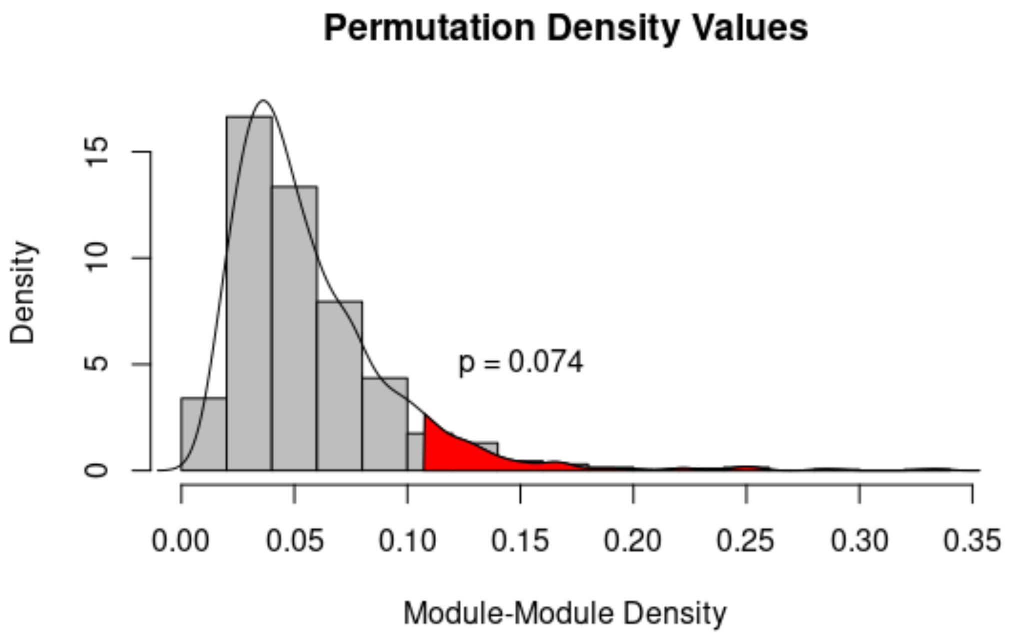

Supplementary Figure 15

Location of *M.tb* epitope variants based on the H37Rv reference genome. Perfectly correlated variants are grouped into SNP sets and labeled with different colors.

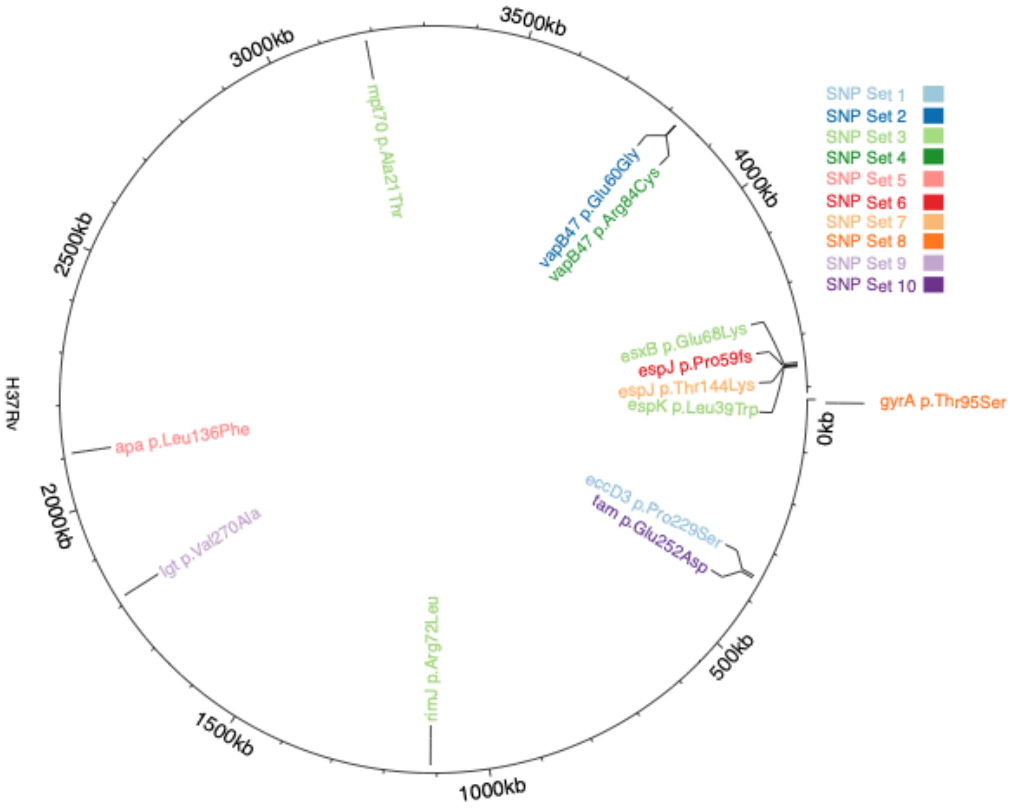

Supplement: Supplementary file 1 — Supplementary Material 1. [file 12920_2025_2164_MOESM1_ESM.pdf]
